# Draft Genome Sequence of *Halomonas* sp. Strain KAO, a Halophilic Mn(II)-Oxidizing Bacterium

**DOI:** 10.1128/MRA.00032-21

**Published:** 2021-02-25

**Authors:** Cameron J. Lee, Mitchell H. Wright, Steven R. Bentley, Anthony C. Greene

**Affiliations:** a School of Environment and Science, Nathan Campus, Griffith University, Brisbane, Queensland, Australia; b Leviathan Biosciences, Brisbane, Queensland, Australia; c Griffith University DNA Sequencing Facility, Brisbane, Queensland, Australia; University of Southern California

## Abstract

*Halomonas* sp. strain KAO is an aerobic, Mn(II)-oxidizing, halophilic bacterium. The draft genome of the isolate contains 47 contigs encompassing 3.7 Mb and a GC content of 64.22%. This sequence will provide essential information for future studies of Mn(II) oxidation, particularly under halophilic conditions.

## Abstract (precis)

*Halomonas* sp. strain KAO is an aerobic, Mn(II)-oxidizing, halophilic bacterium. The draft genome of the isolate contains 47 contigs encompassing 3.7 Mb and a G+C content of 64.22%. This sequence will provide essential information for future studies of Mn(II) oxidation, particularly under halophilic conditions.

## ANNOUNCEMENT

The oxidation of manganese (Mn) in terrestrial and aquatic systems is primarily dictated by microbial processes, whereby soluble Mn(II) is transformed into insoluble Mn(III,IV) oxide (1). Under aerobic conditions, bacteria can oxidize Mn(II); however, the purpose for this process remains uncertain (2, 3). While the mechanisms are well understood in freshwater systems at circumneutral pH, to date, no major studies have investigated halophilic Mn(II) oxidation. The aim of the work associated with *Halomonas* sp. strain KAO was to understand the genes associated with Mn(II) oxidation under halophilic conditions.

*Halomonas* sp. strain KAO was first isolated in December 2018 from saline water sediments that were collected at the shoreline of the Coorong, a hypersaline lagoon in South Australia (36°09.802′S, 139°38.910′E) in mid-2018 (stored at 4°C until used). Enrichment cultures were established in Luria-Bertani broth (Miller; Oxoid) supplemented with 1.7 M NaCl and 1 mM MnCl_2_·4H_2_O. The strain was isolated from a positive Mn(II)-oxidizing enrichment using serial streak plating in the same medium fortified with 16 g/liter agar. Colonies positive for Mn(II) oxidation (dark-brown colonies) were subcultured until pure (4). Mn(II) oxidation was verified using the leucoberbelin blue test (1, 3).

For genomic sequencing, strain KAO was grown in Luria-Bertani broth (Miller) supplemented with 1.7 M NaCl on an orbital shaker at 30°C and harvested as an overnight culture. Purified DNA was extracted using individual enzymes and phenol-chloroform separation (5) and prepared for sequence analysis. Short-insert sequencing libraries were prepared by first shearing the DNA using sonication, followed by electrophoresis to retain fragments 800 bp long. Illumina P5/P7 adapter sequences were ligated to the DNA fragment, and paired-end 150-bp sequencing was conducted on the Illumina HiSeq 4000 platform (Omics2view, Germany). This resulted in 8,035,446 reads, equating to 1.21 Gbp of data and 180-fold coverage. Default parameters were used for all software unless otherwise specified. Sequence reads were processed by trimming poor-quality nucleotides, of <Q30, from the start and end in a 4-bp sliding window using Trimmomatic v0.39 (6). Reads that were >100 bp after trimming were retained. Contaminating vector sequences were screened by Bowtie v2.3.5.1 alignment to the UniVec v10.0 database. Subsequently, the remaining 5,892,500 reads were assembled with the *de novo* assembly tool Velvet v1.2.10 (7). Using the short paired-end read parameter, optimal *k*-mers were assessed by performing assembly across multiple lengths, including 31, 51, 71, 91, 111, and 131. Contigs that were greater than 1,000 nucleotides and had a read pair count of 10 or more were retained. Completeness of assemblies was assessed by QUAST v5.0.2 (8), and single-copy orthologs from the *Oceanospirillales* order were assessed through BUSCO v4.1.4 (9). Taxonomic identification was conducted and annotations were performed using Prokaryotic Genome Annotation Pipeline (PGAP) v4.13 (10).

The 131-kmer *de novo* assembly of strain KAO produced 47 contigs encompassing 3,725,077 bp. The assembled genome possessed a GC content of 64.22%. The largest contig was 1,007,327 bp, and the *N*_50_ and *L*_50_ values were 324,953 bp and 4, respectively. Annotation suggested that the assembly contained 3,517 genes, 2 complete rRNAs (1× 5S and 1× 16S) and 63 tRNAs. Furthermore, 41 pseudogenes were identified, of which 23 were due to incomplete coverage. Additionally, two CRISPR arrays were identified. The assembly was identified to be 99.2% complete based on single-copy orthologs. Average nucleotide identity analysis from PGAP suggests that strain KAO does not align closer than 90% with other bacterial genomes, with the highest percent identity to Halomonas halodenitrificans (DSM 735, ATCC 13511) of 86.99%.

### Data availability.

The genome sequence and annotation data for *Halomonas* sp. KAO were deposited in DDBJ/GenBank under BioProject number PRJNA671517, BioSample number SAMN16623250, SRA number SRX9524101, SRR number SRR13077180, and the accession number JADNRL000000000. The version described in this paper is the first version, JADNRL010000000.
